# Merozoite surface protein 1 paralog is involved in the human erythrocyte invasion of a zoonotic malaria, *Plasmodium knowlesi*

**DOI:** 10.3389/fcimb.2023.1314533

**Published:** 2023-12-04

**Authors:** Seong-Kyun Lee, Tuyet Kha Nguyen, Franziska Mohring, Jin-Hee Han, Egy Rahman Firdaus, Sung-Hun Na, Won-Sun Park, Robert W. Moon, Eun-Taek Han

**Affiliations:** ^1^ Department of Medical Environmental Biology and Tropical Medicine, School of Medicine, Kangwon National University, Chuncheon, Gangwon-do, Republic of Korea; ^2^ Department of Infection Biology, Faculty of Infectious and Tropical Diseases, London School of Hygiene and Tropical Medicine, London, United Kingdom; ^3^ Department of Obstetrics and Gynecology, School of Medicine, Kangwon National University, Chuncheon, Gangwon-do, Republic of Korea; ^4^ Department of Physiology, School of Medicine, Kangwon National University, Chuncheon, Gangwon-do, Republic of Korea

**Keywords:** malaria, *Plasmodium knowlesi*, PkMSP1P, invasion, erythrocyte, CRISPR/Cas9

## Abstract

The zoonotic malaria parasite *Plasmodium knowlesi* is an important public health concern in Southeast Asia. Invasion of host erythrocytes is essential for parasite growth, and thus, understanding the repertoire of parasite proteins that enable this process is vital for identifying vaccine candidates and how some species are able to cause zoonotic infection. Merozoite surface protein 1 (MSP1) is found in all malaria parasite species and is perhaps the most well-studied as a potential vaccine candidate. While MSP1 is encoded by a single gene in *P. falciparum*, all other human infective species (*P. vivax*, *P. knowlesi*, *P. ovale*, and *P. malariae*) additionally encode a divergent paralogue known as MSP1P, and little is known about its role or potential functional redundancy with MSP1. We, therefore, studied the function of *P. knowlesi* merozoite surface protein 1 paralog (PkMSP1P), using both recombinant protein and CRISPR-Cas9 genome editing. The recombinant 19-kDa C-terminus of PkMSP1P (PkMSP1P-19) was shown to bind specifically to human reticulocytes. However, immunoblotting data suggested that PkMSP1P-19-induced antibodies can recognize PkMSP1-19 and vice versa, confounding our ability to separate the properties of these two proteins. Targeted disruption of the *pkmsp1p* gene profoundly impacts parasite growth, demonstrating for the first time that PkMSP1P is important in *in vitro* growth of *P. knowlesi* and likely plays a distinct role from PkMSP1. Importantly, the MSP1P KO also enabled functional characterization of the PkMSP1P-19 antibodies, revealing clear immune cross-reactivity between the two paralogues, highlighting the vital importance of genetic studies in contextualizing recombinant protein studies.

## Introduction

Malaria is one of the most important neglected infectious diseases, causing more than 600,000 deaths worldwide yearly, with 90% of death cases occurring in sub-Saharan Africa areas (Organization WH, 2022). Although previously misdiagnosed as *Plasmodium malariae*, *P. knowlesi* is now increasingly recognized as a common cause of clinical malaria in some Southeast Asia countries for the last decade (Lee et al., 2009; Singh and Daneshvar, 2013; Shearer et al., 2016; Cooper et al., 2020). In 2021 alone, 3,575 known cases were reported in Malaysia with mild to life-threatening symptoms leading to 13 deaths—highlighting that *P. knowlesi* malaria could become an unpredictable risk (Organization WH, 2022). While *P. falciparum* and *P. vivax* were nearly eliminated in Sabah, *P. knowlesi* reached the highest incidence in 2017 (Cooper et al., 2020). Thus, despite clear progress in elimination of transmission between humans, new tools and approaches will be required to mitigate zoonotic parasite transmission.

Merozoites of malaria parasites recognize the erythrocyte membrane *via* a repertoire of receptor–ligand interaction to enable red blood cell (RBC) invasion (Chitnis, 2001). While some of these interactions are conserved across all species, most of the early recognition steps, particularly those that define the host cell tropism, are less well conserved across the different species. Even though many of these processes are most well studied in *P. falciparum*, this species is phylogenetically divergent from other human infective species. In contrast, *P. knowlesi*, the only other human infective species that can be maintained in culture with host cell, human RBCs, clusters with *P. vivax* and the other human infective species and indeed shares some key invasion pathways. For example, *P. vivax* Duffy binding protein (PvDBP) is important for human infection and has a direct orthologue in *P. knowlesi* PkDBP-alpha (PkDBPα) that is also essential for human RBC invasion, with two additional paralogues unique to *P. knowlesi* PkDBP-beta (PkDBPβ) and -gamma (PkDBPγ) providing specific ligands for invasion of primate RBCs (Adams et al., 1990; Waters et al., 1993; Singh et al., 2005). However, despite its importance as a model for non-falciparum invasion still relatively, few *P. knowlesi* antigens have been identified as vaccine candidates (Semenya et al., 2012; Moon et al., 2016).

The merozoite surface protein (MSP) is the most abundant surface protein involved in merozoite binding, egress, and invasion into erythrocytes during the erythrocytic cycle (Bozdech et al., 2003; Moss et al., 2012; Das et al., 2015). MSP1 has been the most well-studied protein in the MSP family, and the protein is segmented by two steps of proteolytic cleavage, resulting in four major polypeptides (MSP1-83, MSP1-30, MSP1-38, and MSP1-42) that form a non-covalent complex on merozoite surface for erythrocyte initial interaction (Blackman et al., 1990; Blackman et al., 1991). Furthermore, the MSP1-42 undergoes a subsequent cleavage step into 33-kDa (MSP1-33) and 19-kDa (MSP1-19) fragments, consisting of a conserved C-terminus detected in the ring stages, whereas the N-terminus of MSP1 is shed out during merozoite invasion (Holder and Freeman, 1984; Holder et al., 1992). MSP1 is immunogenic, exposed at the merozoite surface, and the MSP1-19 domain particularly has been recognized as a potential vaccine candidate. While only a single homologue of MSP1 is present in *P. falciparum*, all other human infective species, including both *P. vivax* and *P. knowlesi*, contain an MSP1 paralog (MSP1P) in their genome. This gene sits directly adjacent to MSP1 in the genome and likely originally arose from segmental duplication. The amino acid sequence analysis showed that MSP1P and MSP1 are similar in structure, in which the C-terminal region has a putative GPI-anchored motif and cysteine residues in two epidermal growth factor (EGF)-like domains (Ahmed et al., 2018a). Previous studies using recombinant protein reveal that *P. vivax* MSP1P (PvMSP1P) has specific binding activity to human reticulocytes, which can be inhibited by monoclonal antibodies against the C-terminus of MSP1P (MSP1P-19), suggesting that the protein plays a role during erythrocyte invasion (Cheng et al., 2013; Han et al., 2018; Han et al., 2019). Although little is known about merozoite surface proteins of *P. knowlesi*, a previous study found that the C-terminus of PkMSP1P is highly conserved in field isolates (Ahmed et al., 2018b). Therefore, evaluating the erythrocyte binding, invasion, and antibody function of PkMSP1P is timely.

The successful establishment of an *in vitro* cultivation system for *P. knowlesi* provides the means to study the biology of the parasite and the ligands involved in the erythrocyte invasion (Kocken et al., 2002; Lim et al., 2013; Moon et al., 2013). Taking that advantage, we first characterized PkMSP1P using recombinant protein and polyclonal antibodies. Using clustered regularly interspaced palindromic repeats (CRISPR)/Cas9-mediated gene knockout/disruption of *pkmsp1p* in the genetically amenable *P. knowlesi* culture system, we were able to demonstrate that while MSP1P has an important and distinct role to MSP1, antibodies against the two cross-react, confounding efforts to study this protein using recombinant systems alone.

## Materials and methods

### *In situ* analysis of *pkmsp1p*

Gene sequence data and protein domain information of *pkmsp1-19* (accession nos. PKNH_0728900 and XP002258582) and *pkmsp1p-19* (PKNH_0728800 and XP002258581) with orthologous genes were obtained from the PlasmoDB website and GenBank, respectively. The amino acid sequence data of *P. knowlesi* MSP1-19, MSP1P-19, and other *Plasmodium* species were aligned using the CLUSTAL-W program in MegAlign Lasergene software ver 7.0 (DNASTAR, Madison, WI).

### Rhesus macaque blood and cord blood samples

Rhesus macaque (*Macaca mulatta*) blood was provided by the National Primate Research Center (NPRC) in the Korea Research Institute of Bioscience and Biotechnology (KRIBB) of Korea. The rhesus macaque blood and human umbilical cord blood samples were collected in a 10-ml sodium heparin tube (BD Vacutainer, Franklin Lakes, NJ). All experiments were performed under relevant guidelines and regulations, and the study was approved by the Kangwon National University Hospital Ethical Committee (IRB no. KNUH-B-2021-06-034). Human umbilical cord samples were obtained from the by-product of routine clinical diagnostic procedures of healthy donors. The written informed consent was obtained from all subjects.

### Expression and purification of recombinant protein

Genomic DNA from the mixed stage of *P. knowlesi* A1-H.1 culture was extracted with QIAamp DNA Blood Mini Kit (Qiagen, Hilden, Germany) and used as a PCR template. The PkMSP1-19- and PkMSP1P-19-tagged glutathione S-transferase (GST) were expressed and purified in an *Escherichia coli* system as follows. Segments of *pkmsp1p-19* and *pkmsp1-19* were amplified using a high-fidelity KOD-plus Kit (TOYOBO, Osaka, Japan) with the following pairs of gene-specific primers: *pkmsp1p-19* forward 5′-ggatccccag *gaattc* atGATCGTGTGAAAAATAACTGCAGA-3′ and reverse 5′-gatgcggccg *ctcgag* GCACACGACCCCCTCATATA-3′; *pkmsp1-19* forward 5′-ggatccccag *gaattc* atTACAGATACTTGGACGGAACGG-3′ and reverse 5′-gatgcggccg *ctcgag* GCTACAGAAAACTCCCTCAAAAAG-3′. Small letters indicate the plasmid-derived sequence for In-Fusion cloning (Clontech, Kusatsu, Japan), and restriction enzymes, *EcoR*I and *Xho*I, are indicated as italicized and underlined letters. The amplicons were cloned into the pGEX-4T-2 expression vector (GE Healthcare, Wauwatosa, WI) by the In-Fusion cloning method, and the sequences of cloned plasmids were confirmed. The positive clone was cloned to recombinant proteins induced with 0.1 mM isopropyl-β-D-thiogalactopyranoside (IPTG, Sigma-Aldrich, St. Louis, MO) and purified using glutathione sepharose 4B resin (GE Healthcare) following the manufacturer’s protocol with the modified elution buffer (50 mM HCl–Tris, 20 mM reduced glutathione, 300 mM NaCl, 2% glycerol, and 200 mM imidazole, pH 8.0) (Spielmann et al., 2003).

### Animal immune sera production and IgG purification

Female BALB/c mice (6 weeks old; Dae Han Bio Link Co., Korea) and Japanese white rabbits were immunized with recombinant PkMSP1P-19 and PkMSP1-19 proteins to generate polyclonal antibodies. In total, 20 μg or 250 μg of recombinant protein was injected with Freund’s complete adjuvant (Sigma-Aldrich), followed by Freund’s incomplete adjuvant (Sigma-Aldrich) three times at a 3-week interval into mouse or rabbit, respectively, and the sera were collected at 2 weeks after the final boost. Total IgGs were purified from 1 ml immune rabbit serum using a protein G HP column following the manufacturer’s protocol (GE Healthcare). The eluted immunoglobulin G (IgG) fractions were dialyzed to incomplete RPMI 1640 (Invitrogen/Gibco, Grand Island, NY) or phosphate-buffered saline (PBS) using centrifugal devices with a 30-kDa cutoff value (Millipore, Billerica, MA). Purified IgGs were pre-adsorbed with human O^+^ erythrocytes (25 μl of RBCs per 4 mg of IgGs) for 30 min to remove any non-specific inhibitory effect (Muh et al., 2018a).

### SDS-PAGE and immunoblotting analysis

Recombinant proteins were analyzed by 13% SDS-PAGE under reducing conditions. The separated proteins were transferred onto a 0.45-μm PVDF membrane (Millipore) in semidry transfer buffer (50 mM Tris, 190 mM glycine, 3.5 mM SDS, 20% methanol) at a constant 400 mA for 40 min using a semidry blotting system (ATTO Corp., Tokyo, Japan). The PVDF membrane (Cytiva, Marlborough, MA) containing recombinant protein was blocked with 5% skim milk in PBS-T (0.5% v/v Tween-20 in 1× PBS) and then incubated with anti-GST antibody (Novagen, Reno, NV) and mouse and rabbit immune sera diluted 1:2,000 in PBS-T. After the primary antibody reaction, the membrane was incubated with the secondary IRDye^®^ goat anti-mouse (1: 5,000 dilution) or IRDye^®^ goat anti-rabbit (1: 5,000) (Li-COR^®^ Bioscience, Lincoln, NE) antibodies to detect antigens. The results were visualized in the Odyssey infrared imaging system and analyzed with Odyssey software (Li-COR^®^ Bioscience) (Lee et al., 2016).

### Reticulocyte enrichment from cord blood

Reticulocytes were enriched from heparinized umbilical cord blood with 19% Nycodenz solution (Accurate Chemical & Scientific Co. Carle Place, NY) in high-KCl buffer using gradient centrifugation as described previously (Sorette et al., 1992). Briefly, fresh cord blood was washed with incomplete RPMI 1640 medium, and leukocytes were removed by Acrodisc^®^ white blood cell syringe filter (Pall Life Sciences, Port Washington, NY). The packed cells were resuspended in high KCl buffer (pH 7.4) and incubated at 4°C for 3 h while rotating. Five milliliters of the RBC-high KCl buffer mixture was overlaid on 3 ml of 19% Nycodenz solution and centrifuged at 3,000 ×g for 30 min. Reticulocytes were harvested from the interface layer, and the concentration and purity were calculated by observing more than 2,000 RBCs in thin blood smear samples stained with new methylene blue stain solution (Sigma-Aldrich).

### Protein binding assay by flow cytometry

The flow cytometry-based direct-binding assay with 5 × 10^5^ RBCs from rhesus macaque or human in 200 μl of incomplete RPMI 1640 medium (Invitrogen/Gibco) was carried out as described previously (Ito et al., 2013). Briefly, RBCs were washed three times with incomplete RPMI 1640 medium and incubated with 0 to 40 µg/ml of GST-tagged recombinant proteins for 4 h at 25°C. Region II of PkDBPα (PkDBPα-RII) and GST-His were used as the positive and negative controls, respectively. After incubation, RBCs were washed twice with phosphate-buffered saline containing 1% BSA (PBS-BSA) and incubated with Alexa Fluor 647-conjugated mouse anti-GST monoclonal antibody (Cell Signaling, Danvers, MA) at 4°C for 1 h in the dark. The samples were washed three times with PBS-BSA and incubated with the thiazole orange (TO) Retic-COUNT reagent (BD Biosciences, Franklin Lane, NJ) for 30 min at 25°C in the dark. A total of 100,000 events were analyzed per sample using FACS Accuri™ C6 Flow Cytometer (Becton-Dickinson, Franklin Lakes, NJ) using FlowJo (v10.9.0, LLC, Ashland, OR). Unstained cells and cells with single TO staining were used for gating the normocytes and reticulocytes, respectively.

### *In vitro* cultivation of blood-stage parasites and parasite protein extraction for western blot

Asexual stage parasites of *P. knowlesi* A1-H.1 were maintained with fresh human erythrocytes in RPMI 1640-based complete medium (Invitrogen Life Technologies, Grand Island, NY) with 10% horse serum as described previously (Moon et al., 2013). To make a homogenous stage, the culture with the mixed stage was synchronized to generate protein extracts using 5% D-sorbitol (Sigma Aldrich) treatment. Ten percent parasitemia of mature schizont-enriched red bood cells was treated with 0.15% saponin (Sigma Aldrich) for 1 min on ice to lyse the RBC membrane. The pellet was washed twice with ice-cold PBS containing 1× protease inhibitor, mixed 1:1, and boiled with reducing buffer. Extracts were separated on 13% polyacrylamide gel and then blotted onto the PVDF membrane following the immunoblotting protocol. Membranes were probed with total IgG from PkMSP1-19- and PkMSP1P-19-immunized rabbits at 1 µg/ml as primary antibodies.

### Indirect immunofluorescence assay

To confirm the localization of PvMSP1-19 and PkMSP1P-19, blood smears for indirect immunofluorescence assay (IFA) were prepared as described previously (Li et al., 2012). Slides smeared with *P. knowlesi* schizont-enriched blood were fixed with 4% paraformaldehyde and blocked with 5% skim milk in PBS. Rabbit anti-PkMSP1-19 and mouse anti-PkMSP1P-19 diluted in 1:50 were used as primary antibodies. Alexa Fluor 488-conjugated goat anti-rabbit IgG or Alexa Fluor 546-conjugated goat anti-mouse IgG secondary antibodies (Invitrogen Life Technologies, Carlsbad, CA) and 4′,6-diamidino-2-phenylindole (DAPI; Invitrogen Life Technologies) were used for secondary antibody staining. The slides were mounted with ProLong Gold Antifade reagent (Invitrogen) and visualized under immersion oil using the FluoView^®^ FV1000 Laser Scanning Confocal Imaging System (Olympus, Tokyo, Japan) equipped with a 60× oil objective. Images were captured using the FV10-ASW 3.0 Viewer software (Olympus). The fluorescence graphic containing more than 500 pixels was calculated by ImageJ (NIH, Rockville, MD).

### Invasion inhibition assay

*Plasmodium knowlesi* wild-type (WT) and knockout (KO) strain schizonts enriched by magnet separation (MACS, Miltenyi Biotec, Rhine-Westphalia, Germany) were cultured in a 96-well plate at 100 μl per well (Ribaut et al., 2008). Initial parasitemia and hematocrit were adjusted to 1%–2% and 2%, respectively. Purified anti-PkDBPα-RII (Muh et al., 2018a), PkMSP1P-19, PkMSP1-19, GST-His, pre-immune rabbit IgG (2 mg/ml), and anti-Fy6 monoclonal antibody against DARC (2C3; 25 μg/ml) (Absolute antibody, Wilton, UK) were mixed to a 96-well plate as tested wells. A positive invasion well was set as the parasite’s normal rupture and reinvasion ability. The parasites were incubated at 37°C in a culture chamber for approximately 10 h until newly invaded ring-stage parasites appeared. After centrifugation, the pellets were washed twice with PBS and fixed with 0.05% glutaraldehyde (Sigma-Aldrich) for 10 min. After washing twice again, the red blood cells were stained with SYBR Green I (Sigma-Aldrich) at 0.2× dilution for 10 min and washed twice with PBS. A total of 200,000 events per sample were analyzed with an Accuri C6 flow cytometer (BD Biosciences). Error bars indicate mean ± SEM for duplicate twice measurement.

### Tight synchronization of fully mature schizonts for transfection

Schizonts were enriched with 55% Nycodenz solution in RPMI 1640, followed by a 2-h incubation with 4-[7-[(dimethylamino)methyl]-2-(4-fluorophenyl)imidazo[1,2-a]pyridin-3-yl] pyrimidine-2-amine (compound 2, kindly provided by Dr. Michael Blackman; Francis-Crick Institute, London, UK) to prevent parasite egress (Collins et al., 2013). Compound 2 was removed from the parasite by washing with RPMI 1640 and then incubated with new red blood cells to allow invasion. The ring stage of infected RBCs was purified from the bottom layer after the 55% Nycodenz enrichment method. Newly purified ring-stage parasites were incubated for up to 24 h for maturation. The tightly synchronized schizonts were enriched again with 55% Nycodenz solution, as described above. After compound 2 removal, the parasites were incubated in RPMI 1640 for 15 min to allow initiation of egress, and then 10 μl of packed cells, including the parasites, were used for each transfection.

### Transfection of the parasites and genotyping

The tightly synchronized mature schizont-stage parasites of *P. knowlesi* were transfected using the Amaxa 4D electroporator (Lonza, Basel, Switzerland) and the P3 Primary cell 4D Nucleofector X Kit L (Lonza) following previous reports (Moon et al., 2016). Briefly, a 20-μg repair template and 20 μg pCas9/sg (Mohring et al., 2019) containing sgRNA sequences for *pkmsp1p* were mixed with P3 Primary Cell nucleofector solution, including supplement 1 (Lonza), and transferred to a Nucleocuvette™ Vessel (Lonza), followed by nucleofection with program FP158. Transfected parasites were immediately transferred to complete media with RBCs and incubated at 550 rpm for approximately 30 min at 37°C to allow invasion before transferring to standard culture conditions. After 24 h, transfected parasites were selected by drug pressure with 100 nM pyrimethamine (Sigma-Aldrich), and the medium, including pyrimethamine, was replaced at daily intervals for 5 days. The transfected parasites were cloned out by limiting dilution and confirmed by genotyping with diagnostic primers of extracted genomic DNA (**Supplementary Tables 1**, **2**).

### Two-step nested PCR

PCR-based repair templates were generated by a two-step nested PCR method to fuse the homology regions (HRs) for *pkmsp1p* gene knockout (KO) as described in our previous study (Mohring et al., 2020). Each HR was amplified with at least 25 nucleotides of homologous barcode overhangs at 3′- or 5′-terms for HR1 or HR, respectively. The HRs were then fused by nested PCR. PCR was carried out with CloneAmp (TaKaRa, Kusatsu, Japan) using the following cycle conditions: 35 cycles of 5 s at 98°C, 20 s at 55°C, and 15 s at 72°C. Seven of 50 μl reactions (total 350 μl) of the final templates were pooled, and ethanol precipitated in 10 μl. All primers used for two-step nested PCR are listed in **Supplementary Tables 1** and **2**.

### Bioinformatic analysis for single guide RNA selection

sgRNA candidates were screened using CRISPR RGEN Tools (Bae et al., 2014), and those with a high score of Out-of-frame Score and only one on-target effect were selected for *pkmsp1p* gene KO (**Supplementary Table 3**).

### Real-time PCR

Ten milliliters of cultured parasites with 2% blood were used for mRNA extraction using the AccuPrep Universal RNA Extraction Kit (Bioneer, Daejeon, Korea) following the manufacturer’s manual. cDNA was amplified from 1 μg of extracted RNA using TOPscript RT DryMIX (dT18 plus) (Enzynomics, Daejeon, Korea), and the synthesized cDNAs were used as a template for RT-PCR with GoTaq qPCR master mix (Promega, Madison, WI). RT-PCR was carried out using the following cycles: hot-start polymerase activation of 2 min at 95°C, followed by 40 cycles of 3 s at 95°C, 30 s at 60°C in AriaMx Real-Time PCR system (Agilent, Santa Clara, CA). All primers used for RT-PCR were listed in **Supplementary Table 1**.

### Statistical analysis

The data were analyzed with GraphPad Prism version 9.5.1 (GraphPad Software Inc., San Diego, CA) and Microsoft Excel 2016 (Microsoft, Redmond, WA). Unpaired *t*-test was used to compare the statistical difference values of each group of binding by protein and *P. knowlesi* invasion inhibition result (*p < 0.05; **p < 0.01; ***p < 0.001). Difference value *p* < 0.05 was considered significant.

## Results

### Characterization of PkMSP1P

**Figure 1:**
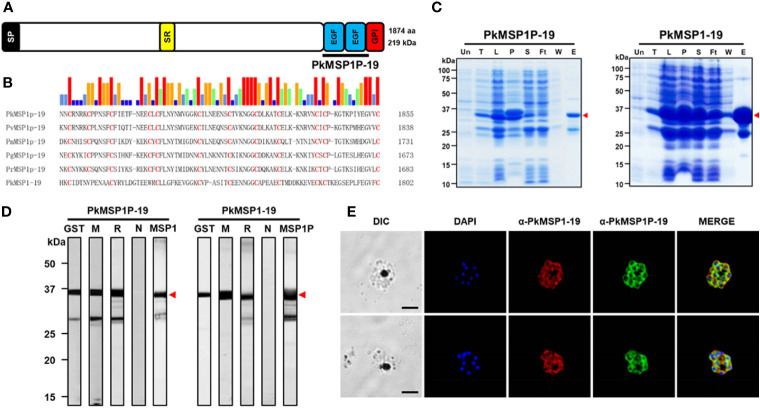
Characterization of PkMSP1P-19 and PkMSP1-19. **(A)** Schematic structure of PkMSP1P (1,875 amino acids (aa)). Indicated are signal peptide (aa 1–36), serine-rich domain (aa 942–970), epidermal growth factor (EGF) domain 1 (aa 1,772–1,807), EGF domain 2 (aa 1,814–1,848), and GPI-anchor (aa 1,852–1,874). PkMSP1P-19 (aa 1,767–1,851) for recombinant protein expression is indicated. **(B)** Amino acid sequence alignment of PkMSP1P-19 with four orthologues and PkMSP1-19. Cysteine residues are highlighted in red, and the similarity of amino acids was indicated as color above alignment. **(C)** SDS-PAGE analysis of the purified recombinant PkMSP1P-19 and PkMSP1-19 with GST-tag. The purified fractions were resolved in SDS-PAGE and stained with Coomassie brilliant blue solution. Un, un-induced fraction; T, total fraction; L, lysate; P, pellet; S, supernatant; Ft, flow-through; W, washing fraction; E, elution. The red arrowhead indicates the expected size of the proteins. A band at ~25 kDa is GST. **(D)** Western-blot analysis of sera with recombinant proteins. GST, mouse anti-GST antibody; M, immune mouse sera; R, immune rabbit sera; N, pre-immune rabbit sera; MSP1 and MSP1P, rabbit anti-MSP1-19 and rabbit anti-MSP1P-19, respectively. One of three mice immunized with the protein was used. Head arrows indicate specific bands. **(E)** The parasites at the late schizont stage are co-labeled with antisera against PkMSP1-19 used as a merozoite surface marker (red color), PkMSP1P-19 (green color), and DAPI for nuclei (blue color). Bars represent 5 μm.

The putative *pkmsp1p* gene (GenBank accession no. XP002258581 and PlasmoDB ID no. PKNH_0728800) is located upstream of *pkmsp1* (XP002258582 and PKNH_0728900, respectively) on chromosome 7 and encodes a 1,874-amino-acid-long protein with predicted high molecular mass (approximately 220 kDa). The predicted primary structure of PkMSP1P consists of signal peptide (aa 1–36), serine-rich domain (aa 942–970), EGF domain 1 (aa 1,772–1,807), EGF domain 2 (aa 1,814–1,848), and GPI-anchor (aa 1,852–1,874) (**Figure 1A**). Orthologues of MSP1P were found in all human infective species except *P. falciparum* (*P. knowlesi*, *P. vivax*, *P. ovale*, and *P. malariae*) and two avian malaria (*P. gallinaceum* and *P. relictum*) according to the PlasmoDB database. Interestingly, the paralogue is also absent among the rodent malaria clade (including *P. berghei* and *P. yoelii*).

Because, in *P. vivax*, only the 19-kDa EGF-like domains of MSP1P (PvMSP1P-19) at the C-terminal region showed binding activity to human red blood cells and can be recognized by malaria-exposed patients (Egan et al., 1995; Cheng et al., 2013; Han et al., 2018), in this study, we focused on its orthologous target in *P. knowlesi*. Sequence alignment of the MSP1P-19 protein showed the conserved 12 cysteine residues across the species, 10 of which are similar in PkMSP1-19 (**Figure 1B**), suggesting that the function of PkMSP1P-19 may be conserved. To address this hypothesis, the recombinant PkMSP1P-19, along with PkMSP1-19 as a control protein, was expressed and purified in soluble form under non-denaturing conditions in bacterial protein expression system, and the purity was assessed by SDS-PAGE. Both proteins migrated as expected molecular weights of approximately 37.2 kDa and 35.3 kDa, and GST protein was observed at 25 kDa (**Figure 1C**, arrowhead). We failed to cleave the GST-tag from the proteins because of precipitation; thus, we generated immune sera from mice and rabbits of the recombinant proteins with the GST-tag. The polyclonal antibodies against PkMSP1P-19 and PkMSP1-19 reacted to their respective recombinant proteins, but no bands were observed with pre-immune sera (**Figure 1D**). However, mutual interaction of the antibody to both proteins was observed, suggesting that antibody cross-reactivity may occur (MSP1 and MSP1P in **Figure 1D**; **Supplementary Figure S1**). MSP1P, like MSP1, is predicted to be attached to the plasma membrane of the parasite via a GPI anchor. The GPI anchor motif requires both an N-terminal signal peptide and also a C-termini GPI anchor motif. The addition of a tag to either the N or C termini would disrupt this signal and remove its GPI anchor. So while tagging of the gene would have additionally helped to understand MSP1P localization, it was not possible without a more complex, internal tagging approach.

An indirect immunofluorescence assay was carried out to determine the localization of the PkMSP1-19 and PkMSP1P-19 in the asexual blood stage of *P. knowlesi* parasites. The fluorescence signal of native proteins was observed, indicating that both proteins are expressed at the mature schizont stage, surrounding the periphery of developing merozoites (**Figure 1E**; **Supplementary Figure S2**). The PkMSP1P-19 antibody gave signal that was similar to that seen for the established merozoite surface marker, MSP1-19; furthermore, free merozoites after rupture were surrounded with the signal. While this suggested that PkMSP1P-19 is present as a merozoite surface protein before and after rupture, the potential cross-reactivity with MSP1-19 meant that no firm conclusions could be drawn.

### PkMSP1P-19 binds to human reticulocytes

**Figure 2:**
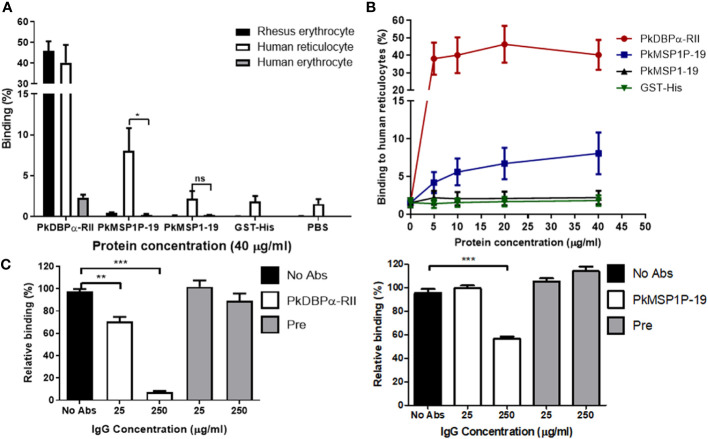
Identification of binding tropism of PkMSP1P-19. **(A)** Comparison of binding affinity of the proteins with rhesus erythrocytes, human reticulocytes, and erythrocytes. 40 μg/mL of the proteins was incubated with erythrocytes. The asterisk indicates statistical significance (*p <* 0.05). **(B)** The binding activity of the proteins to human reticulocytes in a dose-dependent manner. PkDBPα-RII and GST-His were used as positive and negative controls, respectively. **(C)** Inhibition of reticulocyte binding to recombinant proteins by the addition of IgGs to PkDBPα-RII and PkMSP1P-19. 25 or 250 μg/ml of the specific antibodies was treated to the proteins before incubation with reticulocytes. Pre-immune IgG was used as a negative control. ns, not significantly different *p* > 0.05; *, *p* < 0.05; **p < 0.01; ***p < 0.001.

*Plasmodium knowlesi* proteins involved in erythrocyte invasion are critical for determining host cell specificity, and several have distinct binding characteristics between macaque and human erythrocytes. For example, whereas *P. knowlesi* PkDBP-α interacts with both macaque and human erythrocytes, its paralogues, PkDBP-β and PkDBP-γ, only bind macaque erythrocytes (Adams et al., 1992). For those reasons, the binding of PkMSP1P-19 to rhesus macaque and human erythrocytes was investigated using a FACS-based binding assay system. In a previous study, PvMSP1P-19 showed the binding activity to human reticulocytes specifically (Han et al., 2018), so we enriched reticulocytes from human umbilical cord blood and used for reticulocyte binding assay. In the result, PkMSP1P-19 significantly bound to human reticulocytes (8.1% ± 2.8%) but neither macaque erythrocytes nor human erythrocytes. Due to the resource limitation of macaque erythrocytes for the reticulocyte enrichment, it was impossible to specifically test purified macaque reticulocytes. In contrast to PkMSP1P-19, no significant binding activity of PkMSP1-19 to erythrocytes was observed. PkDBPα-RII, a well-known binding partner of the Duffy Antigen Receptor for Chemokines (DARC) on RBCs, showed strong binding activity with human reticulocytes (40.2% ± 8.6%) and erythrocytes (2.3% ± 0.4%) as well as rhesus macaque erythrocytes (46.0% ± 4.6%) (**Figure 2A**), consistent with a previous report (Chitnis and Miller, 1994). We observed a gradual increase in the binding activity of PkMSP1P-19 to human reticulocytes in a concentration-dependent manner (**Figure 2B**), and the binding was significantly inhibited by the purified IgG against PkMSP1P-19 (**Figure 2C**). These results suggest that PkMSP1P-19 binds to human reticulocytes and, therefore, could play a role specifically during invasion. However, in order to mitigate the limitations caused by antibody cross-reactivity, we decided to study the function of PkMSP1P in human erythrocyte invasion by CRISPR-Cas9 genome editing.

### Gene disruption in *P. knowlesi* identifies that PkMSP1P is important for the growth of blood-stage parasites

**Figure 3:**
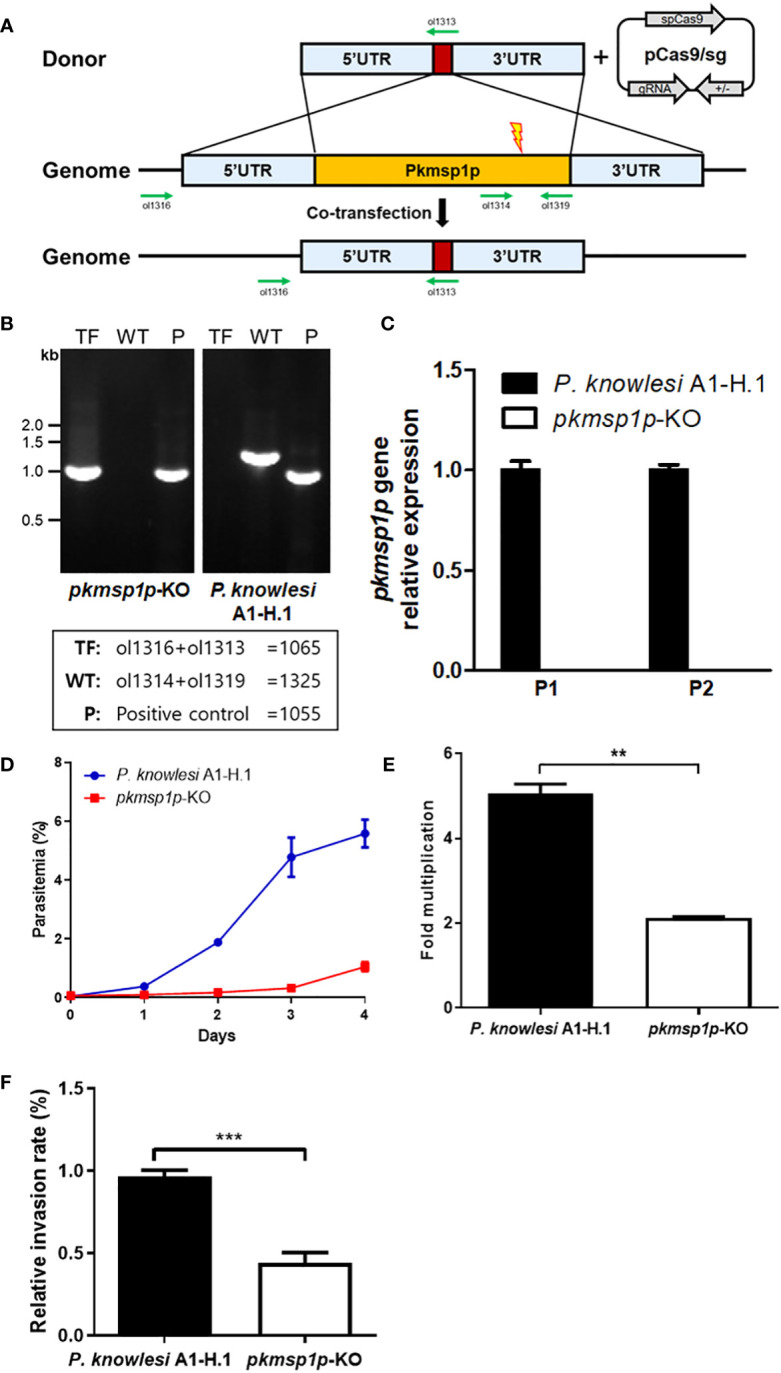
Knockout of *pkmsp1p* impedes the growth of *P. knowlesi*. **(A)** The donor DNA fragment was co-transfected with the pCas/sg construct that confers Cas9 nuclease, sgRNA, and drug resistance for further selection to knockout *pkmsp1p* gene by double crossover. The red box represents the barcode gene that is absent in the *P. knowlesi* genome for genotyping. Green arrows indicate the primers. **(B)** Genotyping of PkMSP1P-KO cloned out by limiting dilution. Genomic DNA was extracted from the parasites, and fragments were amplified with primer pairs indicated in the box. TF, transfected parasites; WT, wild type; P, housekeeping gene (*P. knowlesi* MTIP). **(C)** Real-time PCR to verify the *pkmsp1p* gene after knockout. cDNA was amplified from the mRNA of the *pkmsp1p*-KO line, and *P. knowlesi* A1-H.1 (wild type) served as a template. Two pairs of primers (P1 and 2) targeting the *pkmsp1p* gene were used to detect transcription of the different regions of the *pkmsp1p* gene. The transcription level was calculated based on the housekeeping gene (*P. knowlesi* seryl-tRNA synthetase) level. **(D, E)** Parasitemia and multiplication rate of *pkmsp1p*-KO and the wild type were confirmed. The initial parasitemia was diluted to less than 0.1% and incubated for 4 days. The parasitemia was determined by Giemsa-stained slide under microscopy. **(F)** The enriched schizonts of the wild type and *pkmsp1p*-KO line were incubated with human erythrocytes for 12 h to allow reinvasion, and the invasion rate of the parasites was compared with the wild type. **p < 0.01; ***p < 0.001.

To validate the role of PkMSP1P during the invasion process, gene deletion was carried out using the CRISPR/Cas9 system (Mohring et al., 2019). PCR-based donor DNA to knockout *pkmsp1p* gene was co-transfected with pCas9/sg, which provides spCas9 nuclease, sgRNA, and a positive selection marker (**Figure 3A**). The transfected parasites were cloned out by limiting dilution, and the *pkmsp1p* gene disruption was confirmed by genotyping (**Figure 3B**). This was further confirmed *via* transcriptional analysis of the *pkmsp1p* gene mRNA from wild-type (*P. knowlesi* A1-H.1) and knockout (*pkmsp1p*-KO) parasites, revealing loss of the *pkmsp1p* transcript in KO lines (**Figure 3C**).

We evaluated the parasitemia of *pkmsp1p*-KO parasites compared with the wild type (WT) over 4 days to determine whether the absence of PkMSP1P impacts growth in human erythrocytes. The initial parasitemia of both parasite lines was diluted to be less than 0.1%, and that of the WT parasite line reached up to approximately 6% after 4 days as expected, but the *pkmsp1p*-KO line was just around 1% (**Figure 3D**; **Supplementary Figure S3**), and a comparison of the average fold multiplication rate between WT and KO parasite indicates that growth of the transfected parasite was significantly impaired due to *pkmsp1p* gene disruption with WT averaging fivefold growth and KO only twofold (**Figure 3E**). Furthermore, an “invasion assay” was conducted to specifically determine whether *pkmsp1p* gene disruption affects the conversion of schizonts to the new ring stage in infected human erythrocytes. These results showed that the relative invasion rate of the *pkmsp1p*-KO line was significantly decreased, at around half of the invasion efficiency observed for the WT parasites (**Figure 3F**). These results demonstrated that PkMSP1P is important in one or more of the key processes during the schizont to ring stage transition, which may include steps such as egress, invasion, or early ring development.

### Assessment of anti-PkMSP1P-19-specific IgG potency on knocked-out parasites

**Figure 4:**
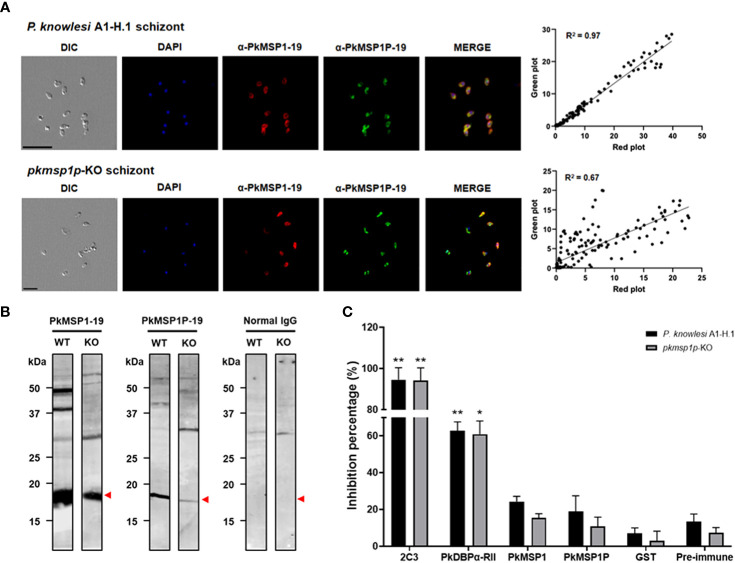
Rabbit polyclonal antibody evaluation on the *pkmsp1p*-KO parasite. **(A)** Co-localization in *pkmsp1p*-KO and wild-type *P. knowlesi* using polyclonal antibodies against PkMSP1-19 or PkMSP1P-19, and then images were captured by confocal microscopy. PkMSP1P-19 rabbit immune sera (red) was dual labeled with mouse immune sera against PkMSP1-19 as a localization marker. The nuclei are visualized with DAPI (blue)—the position and strength comparison between green and red blot from the IFA image. Correlations were calculated using Spearman’s correlation test. **(B)** Parasite lysate Western blot analysis, anti-PkMSP1-19, and anti-PkMSP1P-19 recognized native antigens in *P. knowlesi* PkA1-H.1 (WT) and PkMSP1P-KO (KO) parasite lysates. Normal rabbit IgG was used to detect non-specific bands. Head arrows indicate specific bands. **(C)** Comparison of invasion inhibition activity to *P. knowlesi* PkA1-H.1 and knockout line (*pkmsp1p*-KO) into human erythrocytes. The enriched schizonts were incubated with the antibodies and erythrocytes for 12 h to allow invasion. The inhibition rate was calculated based on the invasion of un-treated wells. Purified IgGs (2.0 mg/ml) from a rabbit immunized with recombinant proteins PkDBPα-RII, PkMSP1-19, PkMSP1P-19, and GST-tag raised antibody for inhibitory baseline, as well as anti-DARC (2C3) monoclonal antibodies (25 μg/ml), were treated to the parasite for invasion inhibition. Significant differences in the effects of pre-immune sera and other antibodies were calculated using an unpaired *t-*test *, *p* < 0.05; **, *p* < 0.01.

Given that the deletion of *pkmsp1p* led to a significant growth defect of *P. knowlesi* merozoites in human RBCs, we next aimed to better validate the specificity of our vaccine-induced polyclonal antibodies against PkMSP1P-19 by testing them on *P. knowlesi* A1-H.1 and *pkmsp1p*-KO lines. To verify that PkMSP1P-19 expression was absent in the KO lines, IFA and immunoblotting with native antigens were performed. It was immediately clear that despite the loss of the MSP1P protein, the signal for the PkMSP1P-19 antibody remained—demonstrating apparent evidence of background cross-reactivity of the antibody (**Figure 4A**; **Supplementary Figure S4**). Despite this, a clear quantitative change in signal was observed. The IFA result was compared using the pixel signal strength of PkMSP1-19 (Red) with PkMSP1P-19 (Green) by two-dimensional scatter data. It showed a high overlap of green and red pixel strength (*R* ^2 =^ 0.97) in the wild type, in clear contrast to the *pkmsp1p*-KO parasite that showed a lower specific signal with a correlation *R* ^2 =^ 0.67 (**Figure 4A**). The subcellular localization assay of *pkmsp1p*-KO with anti-PkMSP1P-19 did not disappear but only decreased the signal compared with WT. We then determined whether antibodies raised against PkMSP1-19 and PkMSP1P-19 can specifically recognize their antigens using WT (*P. knowlesi* A1-H.1) and KO (*pkmsp1p*-KO) schizont-stage parasite lysates; a normal rabbit antibody was used as a negative control (**Figure 4B**; **Supplementary Figure S5**). Both antibodies detected several bands, suggesting that posttranslational modification or proteolytic process of the MSP family commonly occurs in *Plasmodium* proteins (Blackman et al., 1991; Crosnier et al., 2013). The signal strength of the knocked-out strain is weaker than WT in both-antigen detection due to the expression level in the schizont stage being affected after the gene ablation. Although mRNA and DNA level data confirm that we succeeded in knocking out the *pkmsp1p* gene, antibody specificity assays supported the hypothesis that anti-PkMSP1-19 and anti-PkMSP1P-19 antibodies have clear cross-reactivity.

The inhibitory effect of those antibodies during the invasion process into human erythrocytes was evaluated by FACS using PkA1-H.1 and *pkmsp1p*-KO strains [the FACS gating as described previously (Muh et al., 2018b)]. Invasion inhibition of rabbit polyclonal IgGs against PkDBPα-RII, PkMSP1-19, and PkMSP1P-19, as well as monoclonal IgG against human DARC (2C3), was investigated over one replication cycle (24 h). An anti-Fy6 (α-2C3) was used for a positive inhibitory control for the assay with significantly hindered WT and KO line invasion with 94.6% ± 4.2% and 94.2% ± 4.4% [mean ± SEM], respectively. Following, the antibody raised from PkDBPα-RII also showed a high invasion inhibition effect with 62.9% ± 3.3% and 60.9% ± 5.1%, respectively. Comparing pre-immune (PI) sera (13.4% ± 2.9% and 7.3% ± 2.0%) and anti-GST (7.0% ± 2.1% and 2.9% ± 3.8%) invasion inhibition effect with anti-PkMSP1-19 (24.3% ± 2.1% and 15.5% ± 1.6%) and anti-PkMSP1P-19 (18.9% ± 6.0% and 10.7% ± 3.7%), there was no significant difference, except anti-PkMSP1-19 (24.3% ± 2.1%) with anti-GST (7.0% ± 2.1%) observed in WT strain (**Figure 4C**). In conclusion, anti-PkMSP1-19 and anti-PkMSP1P-19 have relatively low inhibitory effects, suggesting that even though PkMSP1P is essential for parasite blood-stage development, the polyclonal antibody specifically targeting PkMSP1P-19 may lack key inhibitory epitopes to block the parasite invasion to human host erythrocytes.

## Discussion

The emerging zoonotic malaria parasite, *P. knowlesi*, with complicated symptoms and high mortality rates, threatens malaria elimination efforts in Southeast Asia (Singh and Daneshvar, 2013). Given that *P. knowlesi* has at least two distinct hosts, determining the host specificity of the repertoire of merozoite surface proteins is vital to understanding the host cell tropism of the parasite (Chitnis and Miller, 1994; Semenya et al., 2012; Dankwa et al., 2016; Moon et al., 2016). Nonetheless, our understanding of the proteins involved in invasion is limited compared with other human infectious malaria species; thus, it is needed to identify more proteins for vaccine or drug development. For these reasons, in this study, we characterized a merozoite surface protein, PkMSP1P, using the recombinant protein and genetic modification method because PvMSP1P is considered a potential novel vaccine candidate with a low polymorphism as well as the naturally acquired humoral immunity (Wang et al., 2011; Cheng et al., 2013; Cheng et al., 2014).

Our previous report that *P. vivax* MSP1P-19 binds to human erythrocytes suggested that the function of the proteins is possibly different from MSP1-19 (Cheng et al., 2013). To demonstrate this suggestion, we decided to study the function of the proteins using *P. knowlesi* because the parasite can be cultivated *in vitro*, unlike *P. vivax*. First of all, we expressed PkMSP1-19 and PkMSP1P-19 recombinant proteins as a soluble form with GST-tag and identified the specific binding of PkMSP1P-19, but not PkMSP1-19 to human reticulocytes, which is consistent with that of *P. vivax* (Han et al., 2018). The different binding activity of the proteins might be due to the extra two cysteine residues in EGF-like domain 1 of MSP1P, which is absent in MSP1 in *P. vivax* and another rodent *Plasmodium* (Carlton et al., 2008), suggesting that the role of PkMSP1P-19 may be distinct from that of PkMSP1-19. We also demonstrated that PkMSP1P-19 does not substantially bind to normocytes of the rhesus macaque. However, we were unable to evaluate the phenotype in reticulocyte binding in macaques due to difficulty in obtaining sufficient material. Comparisons of invasion phenotypes across human and macaque hosts can provide invaluable insight into the meaning of these proteins during the invasion process (Moon et al., 2016), so it is important to validate the function of PkMSP1P in macaque reticulocytes in further study.

The anti-sera against PkMSP1P-19 and PkMSP1-19 recognized the native parasite antigen, and the signal pattern completely merged at the mature schizont and the free merozoite stage in IFAs, demonstrating that PkMSP1P-19 is a merozoite surface protein. However, we also found that anti-PkMSP1-19 and PkMSP1P-19 polyclonal antibodies cross-reacted in the immunoblot data and the non-specific immunofluorescence staining with the KO lines in this study. Because multiple factors could cause this phenomenon, such as polyclonal antibodies sharing the same epitopes on the surface or antibodies induced from the conserved 19-kDa C-terminal region affecting the specificity of MSP family antibodies, a more target-specific approach such as monoclonal or affinity antigen-specific antibodies, will be further studied.

Due to the cross-specific interactions of the antibodies against PkMSP1P-19 and PkMSP1-19, we utilized the gene modification method in *P. knowlesi* (Moon et al., 2013; Hart et al., 2023) to precisely explore whether PkMSP1P was redundant or pivotal to the parasite. We failed to knock out the *pkmsp1* gene (data not shown), suggesting that, despite the presence of a paralogue, MSP1 remains essential for parasite development, as observed in *P. falciparum* (Moon et al., 2016). While work to develop a conditional knockout of *pkmsp1* will be required to confirm this, the apparent essentiality in itself strongly suggests that MSP1 and its paralogue undertake distinct roles in the parasites’ life cycle. In contrast to this, the *pkmsp1p* gene was able to be experimentally deleted. The knockout *pkmsp1p* gene decreased the multiplication and invasion rates compared with WT parasites, suggesting that PkMSP1P is necessary during parasite growth. To further study the role of PkMSP1P, we carried out the invasion inhibition assay that is widely used for assessing functional antibodies for blocking the invasion of merozoites after schizont-stage parasite rupture. Unlike our expectation, however, the IgGs against PkMSP1-19 and PkMSP1P-19 did not show significant invasion inhibitory activity compared with the negative control. This phenomenon was also observed in a previous study (Muh et al., 2020) in which anti-PvMSP1-19 polyclonal antibodies strongly recognized native and recombinant proteins but showed the relatively low inhibitory activity of *P. knowlesi* to the human erythrocytes. This finding brings out the need to use parasitological assays when investigating potential neutralizing antibodies. While these antibodies can clearly block recombinant MSP1P-19 binding to erythrocytes, it is possible that the target epitopes are simply inaccessible in the more complex context of the parasite surface. In addition, like other *Plasmodium* species, MSP1-19 from *P. knowlesi* possesses a distinct charge distribution; all charged residues are accessible on the surface without buried ion pairs (Garman et al., 2003). Therefore, the minor change in charge or hydrophobic residues may lead to a modification in epitope recognition affinity and avidity to the antibody, resulting in the loss of the invasion inhibition ability of the epitope binding site. In these paralogs, except for their C-terminal regions, different domain structures of the proteins could imply different functional roles for each.

MSP1P, like MSP1, is predicted to be attached to the plasma membrane of the parasite *via* a GPI anchor. The GPI anchor motif requires both an N-terminal signal peptide and also a C-termini GPI anchor motif. It is for this reason that work on the very well-studied PfMSP1 has also been carried out using antibodies and not *via* tag approaches. While the C-terminal GPI anchor motif precludes us from undertaking C-terminal epitope/fluorescent tagging of the protein, the introduction of an internal tag or after the N-terminal signal peptide may also provide a route to explore the localization of MSP1P.

This work also highlights the strength of genetic studies in the validation of antibodies—with our MSP1P-KO unequivocally identifying cross-reactivity and off-target activity of our polyclonal MSP1P-19 antibodies. A wider application of the *P. knowlesi* knockout and more recent development of conditional knockouts will provide an important tool for further validation of the numerous *P. vivax* reagents that have been developed with limited access to parasitological assays. Even though there is no significant inhibition of *P. knowlesi* invasion with anti-PkMSP1P-19, the deletion of *pkmsp1p* led to severe growth deficiency of blood-stage *P. knowlesi* parasites, demonstrating for the first time that this protein has a distinct role to MSP1. This, along with the apparent essentiality of MSP1, can affirm that the role of PkMSP1P has diverged from its paralogue and does not have functional redundancy with PkMSP1. This has important and positive implications both for the exploration of MSP1P as a novel candidate but also importantly suggests the presence of MSP1P in non-laveranian parasites will not impact our ability to target MSP1 as a vaccine candidate.

## Data availability statement

The original contributions presented in the study are included in the article/**Supplementary Material**. Further inquiries can be directed to the corresponding authors.

## Ethics statement

The studies involving humans were approved by Kangwon National University Hospital Ethical Committee (IRB no. KNUH-B-2021-06-034). The studies were conducted in accordance with the local legislation and institutional requirements. The participants provided their written informed consent to participate in this study. The animal study was approved by Ethical Guidelines for Animal Experiments of Kangwon National University (KW-220620-3). The study was conducted in accordance with the local legislation and institutional requirements.

## Author contributions

S-KL: Conceptualization, Formal Analysis, Writing – review & editing, Data curation, Investigation, Methodology, Validation, Visualization, Writing – original draft. TN: Writing – original draft, Writing – review & editing, Data curation, Investigation, Methodology, Validation, Visualization. FM: Data curation, Methodology, Resources, Validation, Visualization, Writing – review & editing. J-HH: Data curation, Formal Analysis, Methodology, Validation, Writing – review & editing. EF: Methodology, Resources, Writing – review & editing. S-HN: Methodology, Resources, Writing – review & editing. W-SP: Formal Analysis, Methodology, Resources, Writing – review & editing. RM: Conceptualization, Data curation, Formal Analysis, Funding acquisition, Project administration, Resources, Supervision, Validation, Visualization, Writing – original draft, Writing – review & editing. E-TH: Conceptualization, Data curation, Funding acquisition, Project administration, Supervision, Validation, Writing – original draft, Writing – review & editing.

## Supplementary material

Supplementary Figure 1 Western blot data of **Figure 1D** (Left Panel) G, anti-GTS antibody(Ab); M, Mouse-anti-PkMSP1P-19 GST Ab; R, R-anti-PkMSP1P-19_GST Ab; N, Normal rabbit Ab; MSP1, Recombinant MSP1P protein/Rabbit anti-PkMSP1-19 Ab. (Right Panel) G, GTS Ab; M, Mouse-a-PkMSP1-19 GST Ab; R, R-anti-PkMSP1-19_GST Ab; N, Normal rabbit Ab; MSP1, Recombinant MSP1 protein/Rabbit anti-PkMSP1P-19 Ab.

Supplementary Figure 2 Subcellular localization of anti-PkMSP1P-19 in the ruptured merozoites are co-labeled with antisera against PkMSP1-19 used as merozoite surface marker (red color), PkMSP1P-19 (green color), and DAPI for nuclei (blue color). Bars represent 5 μm.

Supplementary Figure 3 Comparison of parasitemia in wild type and PkMSP1P-KO parasite from different clones of **Figure 3D** experiment. The absence of the *pkmsp1p* gene impedes the growth of parasitemia.

Supplementary Figure 4 The parasites at the late schizont stage on wild type and PkMSP1P-KO parasites are labeled with monoclonal antibodies against PvMSP1P-19 (green color) and DAPI for nuclei (blue color). Four monoclonal antibodies, 2AF4-A2, 2AF4-A6, 3BC6-A5, and 3BC6-B12, were used. Bars represent 5 μm.

Supplementary Figure 5 Western blot data of **Figure 4B** WT, wild type; KO, knock out; M, molecular weight size marker.
