# Supplementary figures and images for: Merozoite surface protein 1 paralog is involved in the human erythrocyte invasion of a zoonotic malaria, Plasmodium knowlesi
Source: Front Cell Infect Microbiol. 2023 Dec 4;13:1314533. doi: 10.3389/fcimb.2023.1314533 (PMC10726050; doi:10.3389/fcimb.2023.1314533)

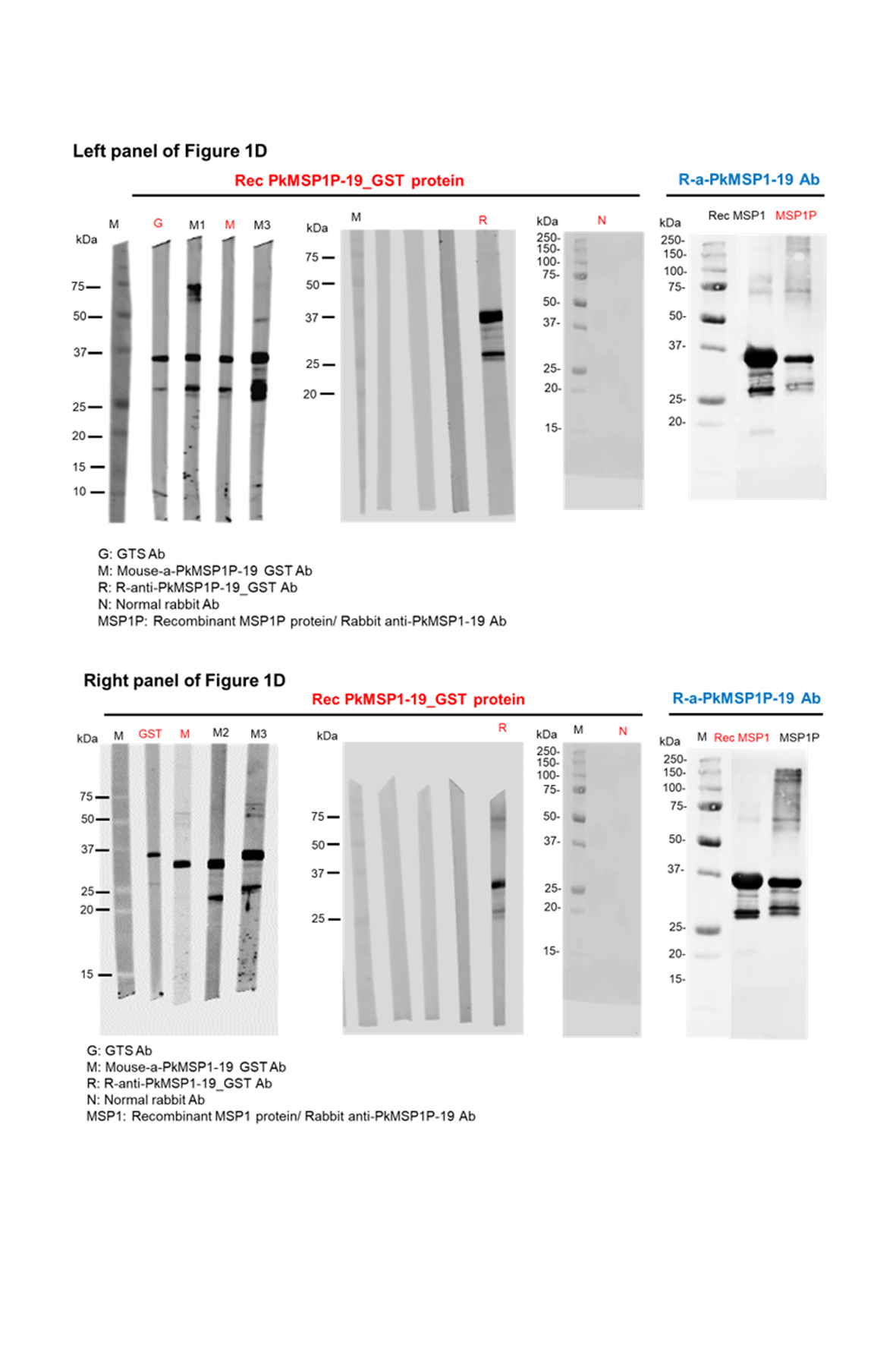

Supplement: Supplementary Figure 1 — Western blot data of Figure 1D (Left Panel) G, anti-GTS antibody(Ab); M, Mouse-anti-PkMSP1P-19 GST Ab; R, R-anti-PkMSP1P-19_GST Ab; N, Normal rabbit Ab; MSP1, Recombinant MSP1P protein/Rabbit anti-PkMSP1-19 Ab. (Right Panel) G, GTS Ab; M, Mouse-a-PkMSP1-19 GST Ab; R, R-anti-PkMSP1-19_GST Ab; N, Normal rabbit Ab; MSP1, Recombinant MSP1 protein/Rabbit anti-PkMSP1P-19 Ab. [file Image_1.tif]

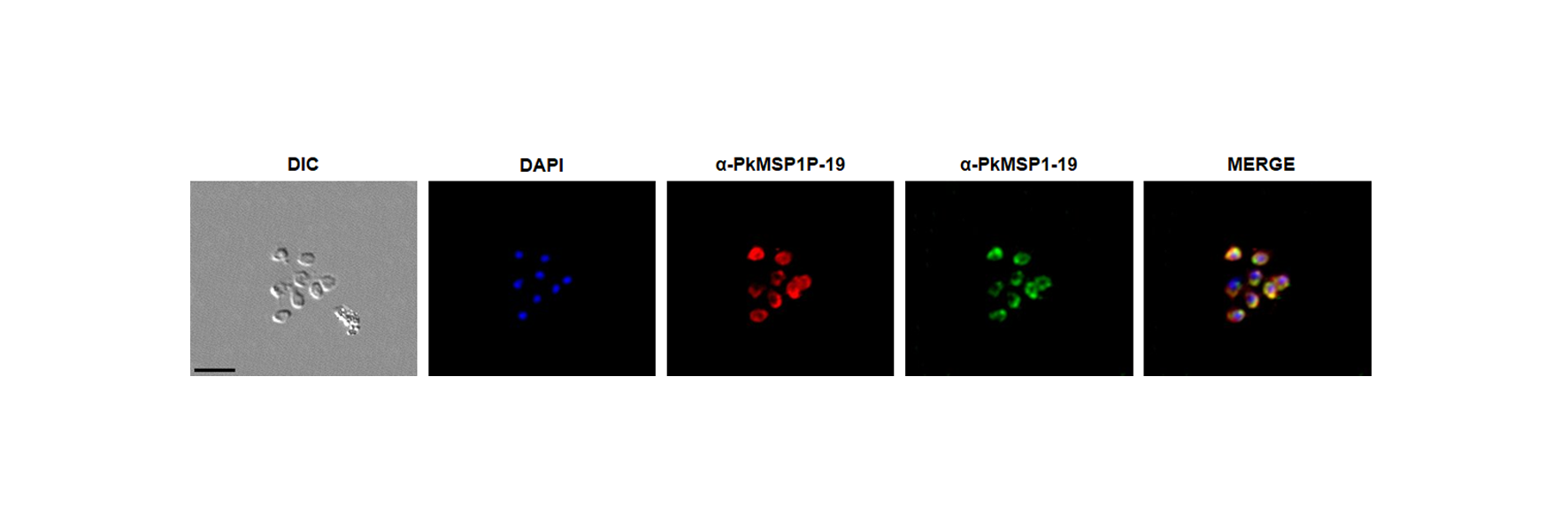

Supplement: Supplementary Figure 2 — Subcellular localization of anti-PkMSP1P-19 in the ruptured merozoites are co-labeled with antisera against PkMSP1-19 used as merozoite surface marker (red color), PkMSP1P-19 (green color), and DAPI for nuclei (blue color). Bars represent 5 μm. [file Image_2.tif]

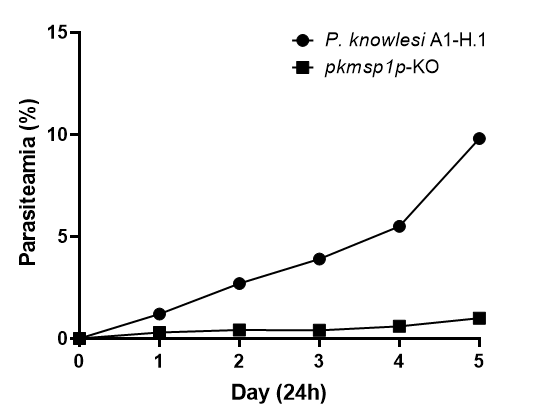

Supplement: Supplementary Figure 3 — Comparison of parasitemia in wild type and PkMSP1P-KO parasite from different clones of Figure 3D experiment. The absence of the pkmsp1p gene impedes the growth of parasitemia. [file Image_3.tif]

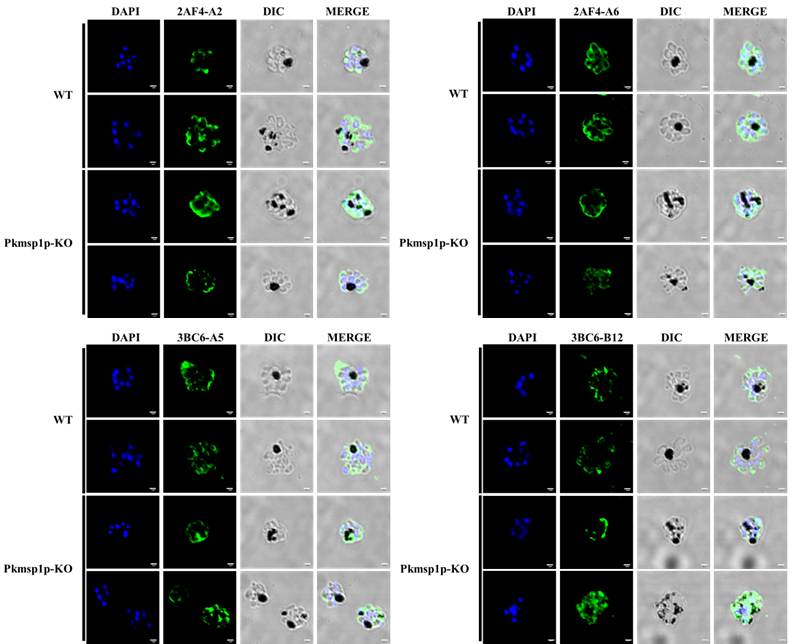

Supplement: Supplementary Figure 4 — The parasites at the late schizont stage on wild type and PkMSP1P-KO parasites are labeled with monoclonal antibodies against PvMSP1P-19 (green color) and DAPI for nuclei (blue color). Four monoclonal antibodies, 2AF4-A2, 2AF4-A6, 3BC6-A5, and 3BC6-B12, were used. Bars represent 5 μm. [file Image_4.tif]

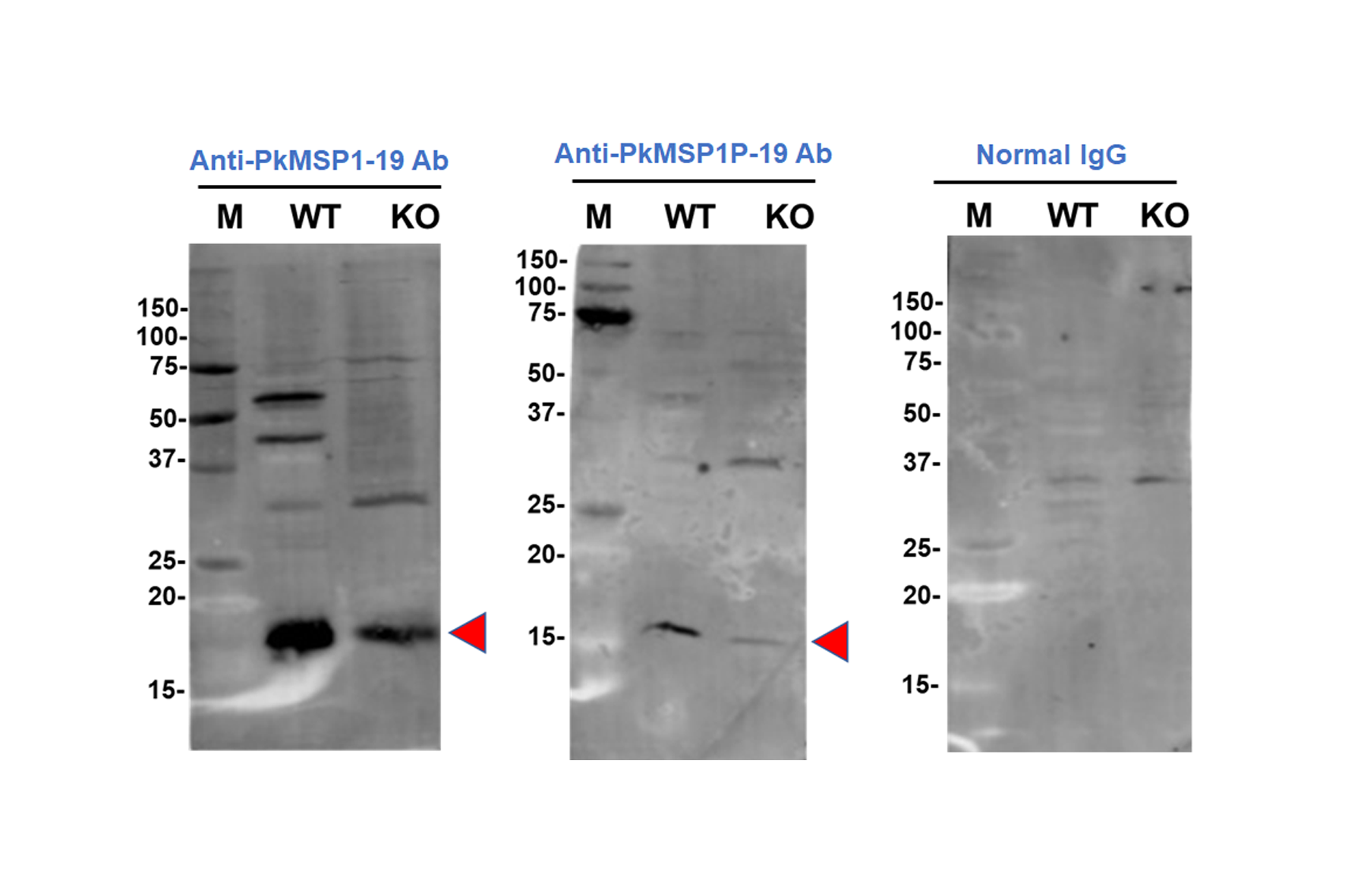

Supplement: Supplementary Figure 5 — Western blot data of Figure 4B WT, wild type; KO, knock out; M, molecular weight size marker. [file Image_5.tif]
