# Supplementary material for: Merozoite surface protein 1 paralog is involved in the human erythrocyte invasion of a zoonotic malaria, Plasmodium knowlesi
Source: Front Cell Infect Microbiol. 2023 Dec 4;13:1314533. doi: 10.3389/fcimb.2023.1314533 (PMC10726050; doi:10.3389/fcimb.2023.1314533)
Supplement: Supplementary file 6 [file Table_1.docx]

**Table S1. Primer sequences in the present study**

| **No.** | **Name** | **Sequence (5'->3')** | **Remark** |
| --- | --- | --- | --- |
| 1 | ol1274 MSP1P HR1 rev | CAGTAGCTCGACTGTAGATCGACTGGCACGTACACACTCACTTC | Preparation of donor DNA for *pkmsp1p* gene knock-out |
| 2 | ol1275 MSP1P HR1 n1 fwd | TGCCCCCTATGCGAATTGGTTCAA |  |
| 3 | ol1276 MSP1P HR2 n1 rev | CACCGCGGAATGGAAGAACCAC |  |
| 4 | ol1277 MSP1P HR2 fwd | CGATCTACAGTCGAGCTACTGAGTGTCTGTGAACTATATTTTTTGTATGTTTGTATG |  |
| 5 | ol1273 MSP1P HR1 n2 fwd | ATATCCGCGGCCTCCGATTTGGTAACCCACTTGA |  |
| 6 | ol1143 PkMSP1P HR2 n1 rev | GAGTTAACATATTTGCCTCCTTCTACCTAG |  |
| 7 | ol1350 PkMTIP_ctag_F | CCCGGGGCGTTTTCGCGTATCTGCGCTTTTTC | Housekeeping gene for genotyping |
| 8 | ol1351 PkMTIP_ctag_R | CCTAGGGGACAATATATCCTCACAGAACAACTTG |  |
| 9 | ol1314 Pkmsp1p19_GT_F | TGGAGAACCTTAACATGTACACCAT | *pkmsp1p* gene detection by PCR |
| 10 | ol1319 Pkmsp1p42_R | GCGGTACCCGGGATCCGCACACGACCCCCTCATATA |  |
| 11 | ol1316 Pkmsp1p_GT_F | GGAGATTGTGCTAAATGTGTACACC |  |
| 12 | ol1313 Pkmsp1pKO_SS | CAGTAGCTCGACTGTAGATCG |  |
| 13 | ol1513 pkmsp1p_RT1_fwd | TACCCACAGCTACATAGCGA | Detection of gene expression by RT-PCR |
| 14 | ol1514 pkmsp1p_RT1_rev | ACCCGAATAGACATCGTCCT |  |
| 15 | ol1515 pksmp1p_RT2_fwd | CGACAACTACGCTGGAATGA |  |
| 16 | ol1516 pkmsp1p_RT2_rev | TGTCTCCCGTTTTGTCCATC |  |
| 17 | ol1517 pkseryl-trna synthetase_fwd | CCACTAGAGGATACCCCTTT | Housekeeping gene for RT-PCR |
| 18 | ol1518 pkseryl-trna synthetase_rev | GGAGTCTACTGAAATGGTCC |  |
| 19 | ol1144PkMSP1P seed 1 fwd | TTACAGTATATTATTCTTAACTATAACATGGTGGGGTTTTAGAGCTAGAA | sgRNA sequence |
| 20 | ol1145 PkMSP1P seed 1 rev | TTCTAGCTCTAAAACCCCACCATGTTATAGTTAAGAATAATATACTGTAA |  |

**Table S2. Primer combination for *pkmsp1p* gene knock-out parasites, and diagnostics**

| **Gene** | **Size of HR1 / HR2 (bp)** | **Primer HR1** | **Primer HR2** | **Primer HR1 + HR2** | **Diagnostic primers** | **Band size (bp)** | **Diagnostic primers** | **Band size (bp)** |
| --- | --- | --- | --- | --- | --- | --- | --- | --- |
|  |  |  |  |  | **WT locus** |  | **Integration locus** |  |
| *pkmsp1p*-KO | 814 / 791 | ol1275 + ol1274 | ol1277 + ol1276 | ol1273 + ol1143 | ol1314 + ol1319 | 1325 | ol1316 + ol1313 | 1065 |

**Table S3. Out-of-frame Score and off-target gene of sgRNAs for *pkmsp1p* in *P. knowlesi* genome**

| **PlasmoDB ID** | **Guide** | **GC Contents (%)** | **Out-of-frame Score** | **Number of found targets** |
| --- | --- | --- | --- | --- |
| PKNH_0728800 | CTTAACTATAACATGGTGGG | 40.0 | 60.2 | 1 |
